# Outbreak of Shiga Toxin-Producing *Escherichia coli* (STEC) O157:H7 Associated with Romaine Lettuce Consumption, 2011

**DOI:** 10.1371/journal.pone.0055300

**Published:** 2013-02-04

**Authors:** Rachel B. Slayton, George Turabelidze, Sarah D. Bennett, Colin A. Schwensohn, Anna Q. Yaffee, Faisal Khan, Cindy Butler, Eija Trees, Tracy L. Ayers, Marjorie L. Davis, Alison S. Laufer, Stephen Gladbach, Ian Williams, Laura B. Gieraltowski

**Affiliations:** 1 Epidemic Intelligence Service Officer, Centers for Disease Control and Prevention, Atlanta, Georgia, United States of America; 2 Division of Foodborne, Waterborne, and Environmental Diseases, Centers for Disease Control and Prevention, Atlanta, Georgia, United States of America; 3 Missouri Department of Health and Senior Services, Eastern District Office, St. Louis, Missouri, United States of America; 4 Communicable Disease Control Services, St. Louis County Department of Health, St. Louis, Missouri, United States of America; 5 Coordinated Outbreak Response and Evaluation Team, Food and Drug Administration, College Park, Maryland, United States of America; University of Maryland School of Medicine, United States of America

## Abstract

**Background:**

Shiga toxin-producing *Escherichia coli* (STEC) O157:H7 is the causal agent for more than 96,000 cases of diarrheal illness and 3,200 infection-attributable hospitalizations annually in the United States.

**Materials and Methods:**

We defined a confirmed case as a compatible illness in a person with the outbreak strain during 10/07/2011-11/30/2011. Investigation included hypothesis generation, a case-control study utilizing geographically-matched controls, and a case series investigation. Environmental inspections and tracebacks were conducted.

**Results:**

We identified 58 cases in 10 states; 67% were hospitalized and 6.4% developed hemolytic uremic syndrome. Any romaine consumption was significantly associated with illness (matched Odds Ratio (mOR) = 10.0, 95% Confidence Interval (CI) = 2.1–97.0). Grocery Store Chain A salad bar was significantly associated with illness (mOR = 18.9, 95% CI = 4.5–176.8). Two separate traceback investigations for romaine lettuce converged on Farm A. Case series results indicate that cases (64.9%) were more likely than the FoodNet population (47%) to eat romaine lettuce (*p-*value = 0.013); 61.3% of cases reported consuming romaine lettuce from the Grocery Store Chain A salad bar.

**Conclusions:**

This multistate outbreak of STEC O157:H7 infections was associated with consumption of romaine lettuce. Traceback analysis determined that a single common lot of romaine lettuce harvested from Farm A was used to supply Grocery Store Chain A and a university campus linked to a case with the outbreak strain. An investigation at Farm A did not identify the source of contamination. Improved ability to trace produce from the growing fields to the point of consumption will allow more timely prevention and control measures to be implemented.

## Introduction

Shiga toxin-producing *Escherichia coli* (STEC) O157:H7 is the causal agent for more than 96,000 cases of diarrheal illness and 3,200 hospitalizations annually in the United States [1]. These preventable illnesses cause substantial morbidity, mortality, and productivity losses. *E. coli* is a bacterium found naturally in the intestines of certain animals including cattle, sheep, and goats. Following a large spinach-associated outbreak of *E. coli* where both feral swine and cattle on or adjacent to the crop fields tested positive for *E. coli* O157, hypotheses emerged on mechanisms of contamination of leafy greens. Ruminants may be vectors involved in direct or indirect fecal contamination (i.e., through contamination of the watershed or irrigation water) of leafy greens or may be sentinel species for *E. coli* in the environment [2], [3]. Human infection occurs an average 3–4 days after bacteria are ingested; symptoms include diarrhea, vomiting, stomach cramps, and a low grade fever lasting for 5–7 days. A rare but severe complication of STEC infections is hemolytic uremic syndrome (HUS), an acute kidney failure that may cause transient or permanent organ damage.

## Materials and Methods

### Ethics Statement

The National Center for Emerging and Zoonotic Infectious Diseases within the Centers for Disease Control and Prevention determined that these investigations did not meet the definition of research as provided by 45 CFR4 6.102(d) and therefore IRB review was not required. The basis for this determination was that the primary purpose of this activity was to identify, characterize, and control disease in response to an immediate public health threat. All participants were explained the purpose of the investigation and participation was voluntary.

### Outbreak Identification

On October 24, 2011 the St. Louis County Department of Health identified a cluster of 17 *E. coli* (STEC) O157:H7 infections. State and local public health officials and the Centers for Disease Control and Prevention (CDC) initiated a multistate outbreak investigation when it was determined that cases resided in multiple counties in the St. Louis metropolitan area on the border of Missouri and Illinois. Using standardized methods, clinical isolates had indistinguishable pulsed-field gel electrophoresis (PFGE) patterns [4]. PFGE patterns from two restriction enzymes (*XbaI* and *BlnI*) were submitted by public health laboratories to PulseNet, the national molecular subtyping network for foodborne disease surveillance. Isolates were also tested by multiple-locus variable-number tandem repeat analysis (MLVA) using standardized protocols at the Minnesota Department of Health or CDC [5].

### Case Definition

Cases were defined as diarrheal illness (3 or more loose stools within a 24 hour period) in a person between October 7, 2011 and November 7, 2011 and carrying the outbreak strain that was confirmed as Shiga toxin-positive (Stx2) based on laboratory testing and indistinguishable from the outbreak strain: PFGE pattern (*XbaI*-pattern EXH01.0047/*BlnI*-pattern EXHA26.0015 or *XbaI*-pattern EXH01.0047/*BlnI*-pattern EXHA26.1381) and an indistinguishable MLVA pattern (Pattern A1), or MLVA unknown [4]. Cases residing outside Missouri with MLVA patterns highly related (up a three repeat difference at a single locus) to the outbreak pattern were only included if they had travel history to Missouri. A primary case was defined as an individual with the earliest onset date in a household. Secondary cases were defined as diarrheal illnesses reported by family members of primary cases more than 24 hours after illness onset of the primary case. Secondary cases were excluded from analyses.

### Case Finding

State and local public health officials in the St. Louis metropolitan area identified laboratory-confirmed cases of STEC O157. Local health care providers were encouraged through the health alert to do testing in patients presenting with diarrhea. PulseNet was used to identify cases matching the PFGE pattern of the outbreak strain submitted to public health laboratories outside of the metropolitan area.

### Hypothesis Generation

During October 28–31, 2011 cases were interviewed with a Standardized National Hypothesis-Generating Questionnaire which was previously developed by CDC in collaboration with local, state, and federal partners. This questionnaire inquires about >200 food and environmental exposures that occurred in the seven days before illness onset. Using a standardized questionnaire ensures that similar exposures are ascertained across many jurisdictions and allows for rapid pooling of data to improve the timeliness of hypothesis-generating analyses. Early investigation by the local health department identified that cases reported common exposures to grocery store chain, Chain A. A supplemental questionnaire was developed that included more than 150 exposures specific to the soup, salad, and hot foods bars at Grocery Store Chain A and travel to or within Missouri.

### Case-control Study

We conducted a matched case-control study using a questionnaire that included exposures previously associated with STEC infections (e.g. ground beef), and exposures reported by ≥50% of cases during hypothesis generation to identify exposures associated with infection in the 7 days before illness onset. Our analyses included 22 cases from Missouri that were identified as of November 2, 2011. Controls were individuals who did not have diarrhea in the month preceding the interview and were geographically matched to cases using a reverse telephone number directory of landline telephones. A list of 90–110 neighborhood control phone numbers was generated for each case, and we aimed to recruit three controls per case. Controls were proportionally recruited by age category to ensure similar distributions of cases and controls in the following age categories: <18 years old, 18–50 years old, >50 years old. Controls were interviewed about their exposures during the third week of October to match the exposure period of cases. All data were analyzed using SAS 9.3 (SAS Institute, Cary, NC) and exact conditional logistic regression models. Matched odds ratios (mOR) with exact 95% Wald confidence intervals are reported.

### Case Series

The case-series study evaluated all cases, including those identified after the completion of the case-control study. Cases were interviewed by state and local health departments with a questionnaire that included exposures previously associated with STEC infections and exposures reported by ≥50% of cases in the case-control study. Reported food exposures among cases were compared with the Foodborne Diseases Active Surveillance Network (FoodNet) Population Survey Atlas of Exposures, 2006–2007 to determine whether foods were eaten more frequently by cases compared to the FoodNet population [6]. Ten state health departments comprised the 2006–2007 FoodNet sites which captures 15% of the United States population and assessed all food exposures in the seven days prior to interview of a randomly chosen subset of individuals who live in the FoodNet catchment area [6]. We compared frequency of consumption of each food among patients in the case series to the FoodNet population using the binomial test. All data were analyzed using SAS 9.3 (SAS Institute, Cary, NC).

### Traceback Investigation

State and local health departments and the Food and Drug Administration (FDA) conducted traceback investigations of implicated food items. FDA inspected a lettuce processor (Processor A) and two farms used to supply Processor A (Farms A and B).

## Results

### Case Finding

**Figure 1:**
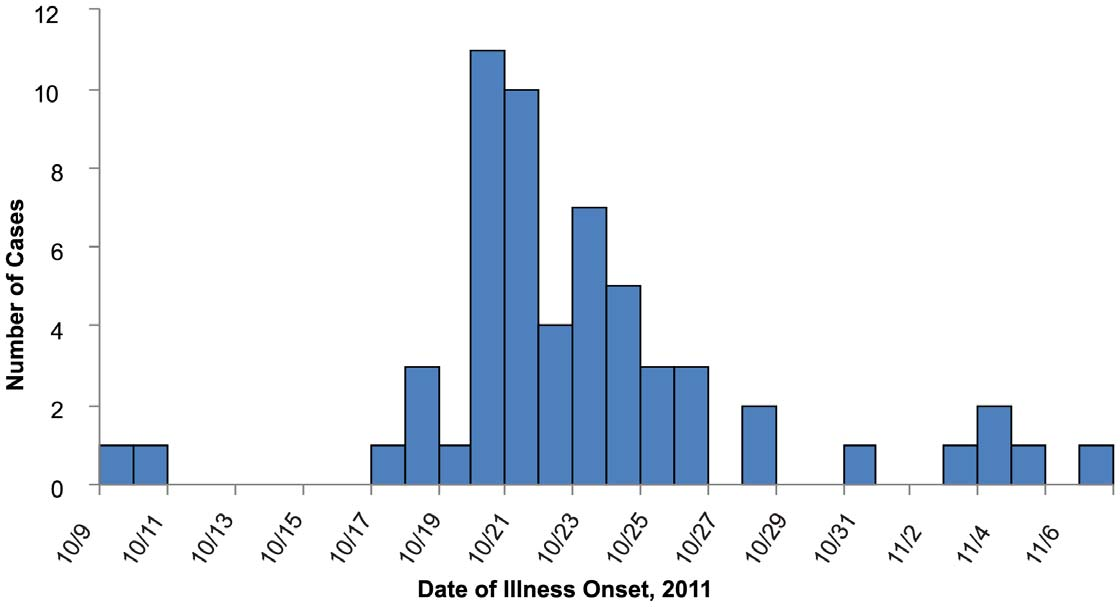
Epidemic curve of infections with the outbreak strain of STEC O157, by date of onset.

**Figure 2:**
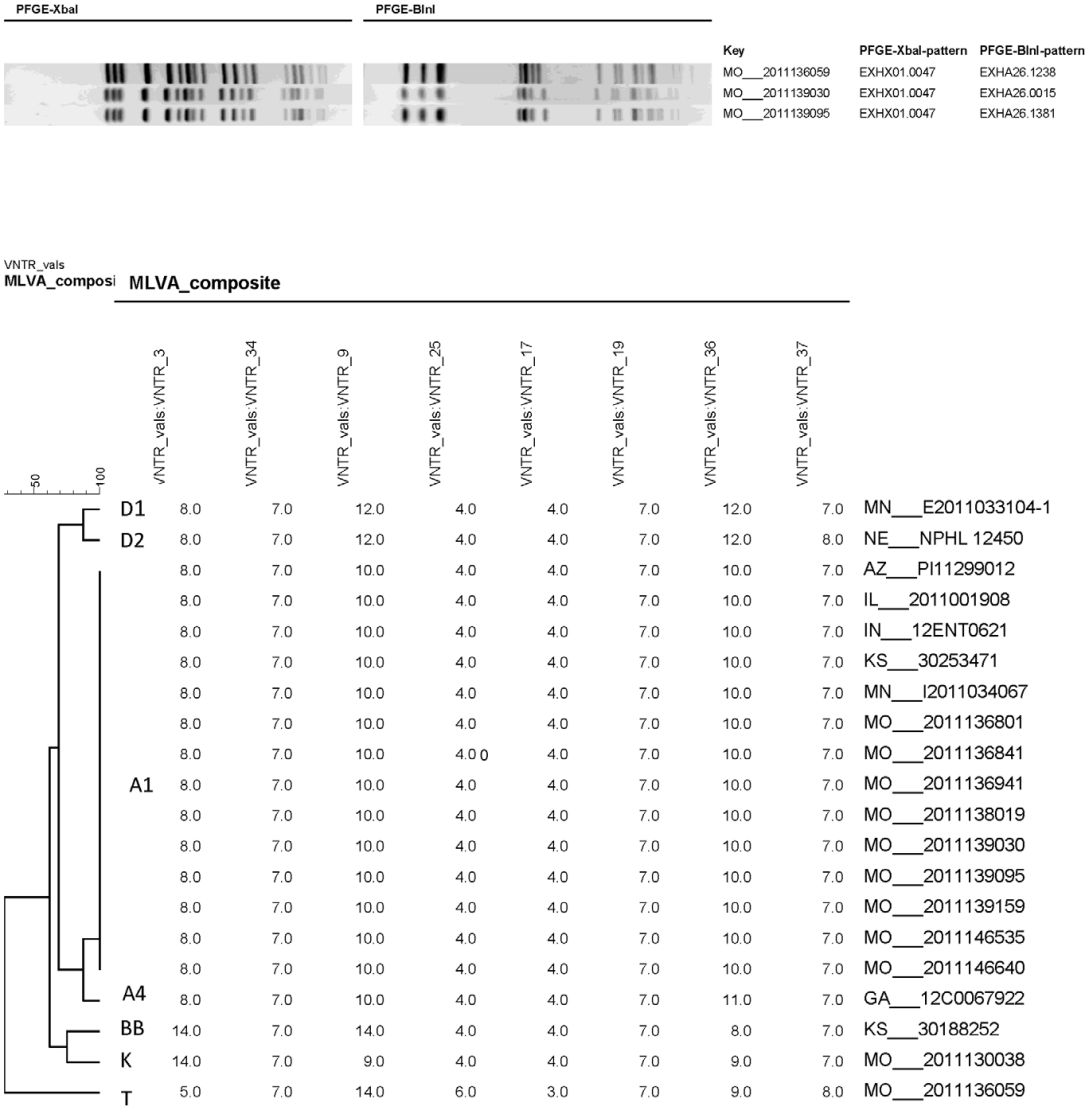
Molecular typing of the outbreak strain of STEC O157. 2a: Representative PFGE *Xba*I and *Bln*I pattern combinations associated with the outbreak investigation. Pattern combination EXHX01.0047/EXHA26.0015 was the main outbreak PFGE pattern. **2b:** Representative MLVA patterns associated with the outbreak investigation. Pattern A1 was the outbreak MLVA pattern.

Overall, 58 cases of STEC in 10 states matching the outbreak strain were identified: Arizona (1), Arkansas (2), Georgia (1), Illinois (9), Indiana (2), Kansas (2), Kentucky (1), Minnesota (2), Missouri (37), and Nebraska (1). Onset dates ranged from October 9 through November 7, 2011 (Figure 1). The median age of cases was 28 years (range 1–94 years); 61% were female. Among 50 cases with information available, 34 (68%) were hospitalized and three cases of 47 with information available (6.4%) developed hemolytic uremic syndrome (HUS). No deaths were reported. Figure 2a depicts the *XbaI* and *BlnI* pattern combinations associated with the outbreak investigation. Figure 2b depicts representative MLVA patterns associated with the outbreak investigation.

### Hypothesis Generation

Between October 24 and October 30, 2011, 14 interviews had been conducted by state and local public health officials. Of those interviewed, 13 (92%) of cases reported any leafy greens and 12 (86%) had consumed romaine lettuce. Ten of fourteen cases (71%) reported eating at any salad bar. Nine of ten cases (90%) who reported eating at any salad bar had eaten at a Grocery Store Chain A salad bar. Grocery Store Chain A estimated that 15% of all shoppers purchase an item from the salad bar based on historic purchase data. Cases also reported high rates of consumption of milk (86%, multiple brands), apples (79%), and tomatoes (79%, multiple varieties).

### Case-Control Study

Twenty-two cases and 82 matched controls were included in the case-control study. On age category-adjusted analysis (<18 years old, 18–50 years old, >50 years old), illness was significantly associated at the significance level of α = 0.05 with Grocery Store Chain A salad bar (matched odds ratio (mOR) = 18.9, 95% confidence interval (95% CI) = 4.5–176.8), any romaine lettuce consumption in the seven days before illness onset (mOR = 10.0, 95% CI = 2.1–97.0), and shopping at Grocery Store Chain A (mOR = 4.1, 95% CI = 1.1–23.0) (Table 1). Illness was not significantly associated with any individual item on the Grocery Store Chain A salad bar, consumption of any other leafy green, or shopping at any other grocery store or salad bar.

**Table 1:** Age category-adjusted matched odds ratios of selected exposures in case patients vs. controls.

Exposure†	Cases(total = 22) n/N (%)	Controls(total = 82) n/N (%)	Matched OddsRatio (95% CI)	*p*-value
Grocery Store Chain A Salad Bar	17/22 (77.3%)	4/80 (5.0%)	18.9 (4.5–176.8)	<0.001
Any Romaine Lettuce	17/20 (85.0%)	32/78 (41.0%)	10.0 (2.1–97.0)	<0.001
Grocery Store Chain A	19/22 (86.4%)	47/82 (57.3%)	4.1 (1.1–23.0)	0.04
Any Spinach	8/21 (38.1%)	19/80 (23.8%)	2.3 (0.7–8.1)	0.21
Any Iceberg Lettuce	10/18 (55.6%)	38/79 (48.1%)	1.9 (0.6–8.0)	0.36
Any Cabbage	3/20 (15.0%)	7/79 (8.9%)	1.9 (0.3–11.2)	0.64
Ground Beef Dish at Home	6/20 (30.0%)	41/79 (51.9%)	0.4 (0.1–1.3)	0.15

† Any exposure in the seven days before illness onset.

### Case Series

Forty case interviews were included in the case series study. Cases (50.0%) were significantly more likely (*p-*value = 0.003) than the FoodNet population (24%) to eat granola bars, breakfast, power, or protein bars, although cases did not report a common variety or brand of bar consumed (Table 2). Cases (64.9%) were significantly more likely than the FoodNet population (47%) to eat romaine lettuce (*p-*value* = *0.013), and 61.3% of cases reported that they consumed romaine lettuce from the Grocery Store Chain A salad bar. Cases (17.1%) were also significantly more likely than the FoodNet population (8%) to eat sprouts other than alfalfa (i.e., bean, clover, broccoli, daikon, radish, etc.) (*p-*value* = *0.028). However, sprout consumption was not significantly associated with illness in the case-control study.

**Table 2:** Frequency of selected case-series exposures in case patients vs. FoodNet population survey exposure.

Exposure†	Cases(total = 40) n/N (%)	FoodNet PopulationSurvey %‡	*p*-Value
Granola bars, breakfast, power, or protein bars	14/28 (50.0%)	24%	0.003
Sprouts other than alfalfa (i.e., bean, clover, broccoli, daikon radish, etc.)	7/41 (17.1%)	8%	0.028
Any Romaine Lettuce	24/37 (64.9%)	47%	0.013
Romaine Lettuce from Grocery Store A Salad Bar	19/31 (61.3%)	n/a	n/a
Sausage	9/40 (22.5%)	18%	0.112
Mexican-style soft cheese (queso fresco, queso blanco)	5/39 (12.9%)	6.4%	0.065
Lamb	2/32 (6.3%)	2%	0.134
Crab, lobster, crayfish	5/32 (15.6%)	9%	0.156
Fresh spinach	10/39 (25.6%)	24%	0.141
Iceberg lettuce	19/35 (54.3%)	46%	0.083
Grocery Store Chain A Salad Bar	27/33 (81.8%)	n/a	n/a

† Any consumption in the seven days prior to illness onset.

‡ Foodborne Diseases Active Surveillance Network (FoodNet) Population Survey Atlas of Exposures, 2006–2007 [6].

### Traceback Investigation and Control Measures

FDA and several state agencies conducted traceback investigations for romaine lettuce to try to identify the source of contamination. Traceback investigations focused on ill persons who had eaten at salad bars at several locations of Grocery Store Chain A and ill persons at university campuses in Minnesota (1 ill person) and Missouri (2 ill persons). Traceback analysis determined that a single common lot of romaine lettuce harvested from Farm A was used to supply the Grocery Store Chain A locations as well as the university campus in Minnesota during the time of case exposure. This lot was also provided to a distributor that supplied lettuce to the university campus in Missouri, but records were not sufficient to determine if this lot was sent to this university campus. An investigation at Farm A did not identify the source of the contamination; however, Farm A was no longer in production at the time of the investigation. Chain A voluntarily removed suspected food items, including romaine lettuce, from the salad bar on October 26, 2011. While no isolate was recovered from the food product samples taken throughout the supply chain, the epidemiological investigation indicated that romaine lettuce was the food vehicle for this outbreak, which provided the direction for the traceback investigation.

## Discussion

A large number of human illnesses have been linked to consumption of leafy greens contaminated with STEC O157 [3], [7], [8], [9], [10], [11], [12]. Although there are multiple routes for human infection with STEC O157, outbreaks associated with leafy greens are becoming increasingly common [7]. The 58 laboratory-confirmed cases of STEC O157 matching the outbreak strain are likely only a fraction of the individuals who became ill as a part of this outbreak. Under-diagnosis results from variations in medical care seeking behaviors, specimen submission, laboratory testing, and test sensitivity (1). Burden of illness calculations, by Scallan et al. (2011), estimate 26.1 unreported infections for every one laboratory-confirmed case of STEC O157 (90% credibility interval: 16.1–41.3) [1]. Extrapolating these under-diagnosis estimates to this outbreak, there were an estimated 1,514 illnesses (90% credibility interval: 934–2,396) associated with this outbreak, although these estimates may have been affected by the media attention surrounding the outbreak in the St. Louis metropolitan area. However, strict microbiological criteria requiring both a two enzyme PFGE match and MLVA match excluded a number of suspected cases who sought medical attention but did not match the outbreak strain.

Based on the epidemiologic and environmental investigations, the likely source of this outbreak was contaminated romaine lettuce sold predominantly at Grocery Store Chain A salad bars in the St. Louis, Missouri metropolitan area. This outbreak demonstrates the important public health aim to decrease the burden of STEC O157 in produce products that are often consumed raw. Instituting policies to help prevent contamination may help prevent future STEC outbreaks.

Consumption of romaine lettuce in the seven days before illness onset was significantly associated with illness in both the case-control and case series studies. Eating alfalfa sprouts was not significantly associated with illness in either study. Sprouts other than alfalfa (i.e., bean, clover, broccoli, daikon radish, etc.) were significantly associated with illness only in the case-series study when comparing cases to the FoodNet population, but were not found to be associated with illness in the case-control study. Seven of 41 cases (17.1%) in the case series reported sprouts in the seven days before illness onset; such a low reporting percentage of diverse sprouts, even if more than expected compared to the FoodNet population, is unlikely to explain all of the cases involved in this outbreak, even accounting for recall error among cases not reporting sprouts. Romaine lettuce consumption accounts for a higher proportion of illnesses (64.9% in case series) and trace-back identified a common supplier linking illness in multiple states.

This study was subject to several limitations including recall bias, small sample size, comparison populations, and selection of a single *E. coli* colony for microbiological testing. For the hypothesis-generating questionnaire and the case-control and case-series study, cases were asked to recall food histories seven days before illness and were interviewed one to four weeks after their illness and controls were asked to recall their food histories approximately two weeks to their interviews. Throughout the investigation there were local media reports implicating different food exposures before all case-patient interviews were completed, potentially biasing case-patients’ food histories. Our comparison group in the case-series study was the FoodNet population from ten FoodNet sites, but does not include individuals from the St. Louis metropolitan area where dietary habits may be different. Additionally, our case-control study sample was limited to case enrollment and not powered to detect a single food item on the salad bar. As for the microbiological investigation, there is always a chance that another strain of O157 serotype may have been missed since MLVA results were unavailable for two isolates with PFGE matches to the outbreak strain and multiple non-sorbitol fermenting colonies typical for O157 serotype are not routinely tested due to a lack of resources. Additionally, strains of non-O157 serotype could have also been missed since laboratories testing primary samples do not routinely pick sorbitol fermenting colonies for testing if they have already identified typical colonies for the non-O157 serotype.

Despite these limitations, our investigation was able to determine that romaine lettuce harvested at Farm A was used to supply both the salad bars at Grocery Store Chain A locations and a university campus in Minnesota. Romaine lettuce served on the salad bars at all locations of Grocery Store Chain A came from a single lettuce processing facility via a single distributor. This indicates that contamination of romaine lettuce likely occurred before the product reached the multiple Grocery Store Chain A locations.

Outbreaks of bacterial foodborne illnesses associated with raw produce are becoming increasingly common and STEC O157 has been isolated from the watershed in a major produce production region in California [3], [7]. Outbreaks associated with raw produce are not likely to subside in absence of changes in agricultural practices. The dynamic contamination of the watershed in the produce-growing Salinas Valley described by Cooley et al. (2007) raises important questions about the development of novel prevention strategies to minimize the likelihood of future leafy green-associated outbreaks [3]. Improved ability to trace produce from the growing fields to the point of consumption will allow more timely prevention and control measures to be implemented. Records indicate that the distributor that supplied lettuce to the university campus in Missouri received lettuce from Farm A, but records were not sufficient to determine if this lot was sent to this university campus. Improved record keeping strategies would aid in food product traceability from farm to fork and ultimately prevent additional illnesses.
